# The inaugural issue of *Neurosurgical Focus: Video*

**DOI:** 10.3171/2019.7.FocusVid.Editorial

**Published:** 2019-07-01

**Authors:** William T. Couldwell

**Affiliations:** Editor in Chief, *Neurosurgical Focus*

Since the advent of video supplements introduced by *Neurosurgical Focus* in 2012, the interest in this educational platform and number of submissions for publication have risen dramatically. Recognizing this need for an enhanced platform for video materials, we have increased our capacity in this arena. Initially published twice per year, our video supplement issues are currently published quarterly.

The growing international interest for this media has demonstrated to the Journal of Neurosurgery Publishing Group (JNSPG) the need for an enhanced platform for its dissemination. In this regard, it is my absolute pleasure to announce the establishment of the inaugural issue of *Neurosurgical Focus: Video*. This new periodical will capitalize on the current technological video enhancements available in an open-access web-based publication. Under the outstanding leadership of *Neurosurgical Focus* Associate Editor Aaron Cohen-Gadol, the new journal will utilize the advantages of video to demonstrate the following themes of operative intervention:

• Operative anatomy
• Surgical technique
• Novel or enhanced surgical approaches
• Surgical complications
• New technology and instrumentation

In addition, in future issues we wish to initiate an operative forum for open feedback and discussion of material published. We intend this new journal to function as a world platform and encourage submissions accordingly. The quarterly format will continue, with topic-specific editors with additional experience in video editing to offer the most contemporary peer review. We invite video submissions that demonstrate both macro- and microscopic and endoscopic techniques.

We hope you enjoy the inaugural issue of *Neurosurgical Focus: Video*! We have compiled a remarkable set of videos that include anatomic correlative enhancements of surgical principles and technique with the management of patients with symptomatic cavernous malformations.

